# Plant Derived Bioactive Compounds, Their Anti-Cancer Effects and *In Silico* Approaches as an Alternative Target Treatment Strategy for Breast Cancer: An Updated Overview

**DOI:** 10.3390/cancers13246222

**Published:** 2021-12-10

**Authors:** Vijayakumar Shrihastini, Pandiyan Muthuramalingam, Sivakumar Adarshan, Mariappan Sujitha, Jen-Tsung Chen, Hyunsuk Shin, Manikandan Ramesh

**Affiliations:** 1 Department of Biotechnology, Sri Shakthi Institute of Engineering and Technology, Coimbatore 641062, Tamil Nadu, India; shrihastinivijayakumar@gmail.com (V.S.); psujitha1325@gmail.com (M.S.); 2 Department of Biotechnology, Science Campus, Alagappa University, Karaikudi 630003, Tamil Nadu, India; sadarshan1999@gmail.com (S.A.); mrbiotech.alu@gmail.com (M.R.); 3 Department of Life Sciences, National University of Kaohsiung, Kaohsiung 811, Taiwan; 4 Department of Horticultural Sciences, Gyeongsang National University, Jinju 52725, Korea; shinpomo@gnu.ac.kr

**Keywords:** breast cancer, triple-negative breast cancer, metastasis, medicinal plants, bioinformatics

## Abstract

**Simple Summary:**

Breast cancer is one of the leading causes of death among women worldwide. Breast cancer may be provoked due to several physical, chemical and environmental factors. Moreover, genetic alternations that are inherited via generations may be a reason for the occurrence of cancer. When the cancer is benign, several therapeutic approaches are available to treat it. In case of malignancy, cancer may spread to other body parts and lead to death. Recent studies focus on the use of indigenous medicinal plants for the treatment of various cancers and particularly breast cancer. This could be an alternative to other treatment methods, as they cause minimal side effects when compared to chemo-drugs. In addition to that, high-throughput omics tools have paved the way for efficient drug targeting, and it would be a promising application for finding the interaction of drug molecules in human systems.

**Abstract:**

Cancer is one of the most common malignant diseases that occur worldwide, among which breast cancer is the second leading cause of death in women. The subtypes are associated with differences in the outcome and were selected for treatments according to the estrogen receptor, progesterone receptor, and human epidermal growth factor receptor. Triple-negative breast cancer, one of the subtypes of breast cancer, is difficult to treat and can even lead to death. If breast cancer is not treated during the initial stages, it may spread to nearby organs, a process called metastasis, through the blood or lymph system. For in vitro studies, MCF-7, MDA-MB-231, MDA-MB-468, and T47B are the most commonly used breast cancer cell lines. Clinically, chemotherapy and radiotherapy are usually expensive and can also cause side effects. To overcome these issues, medicinal plants could be the best alternative for chemotherapeutic drugs with fewer side effects and cost-effectiveness. Furthermore, the genes involved in breast cancer can be regulated and synergized with signaling molecules to suppress the proliferation of breast cancer cells. In addition, nanoparticles encapsulating (nano-encapsulation) medicinal plant extracts showed a significant reduction in the apoptotic and cytotoxic activities of breast cancer cells. This present review mainly speculates an overview of the native medicinal plant derived anti-cancerous compounds with its efficiency, types and pathways involved in breast cancer along with its genes, the mechanism of breast cancer brain metastasis, chemoresistivity and its mechanism, bioinformatics approaches which could be an effective alternative for drug discovery.

## 1. Introduction

Since the start of their existence, human beings have explored a variety of plant species for curing illnesses and improving health [1]. As a result, they have identified a huge array of bioactive compounds with extensive therapeutic potentials in plants. Flavonoids, carotenoids, alkaloids, and phenolics are some of the most highly researched plant chemicals with a wide range of medicinal properties including antitumor activity [2,3,4]. Cancer, the second-most leading cause of mortality, is a genetic disease that is caused due to uncontrolled proliferation of abnormal cells in the body and the metastasis of these cells to other body parts [5], among which breast cancer is one of the most common type. Breast cancer is an example of a heterogeneous disease caused among women. After skin cancer, the second most commonly diagnosed is breast cancer. Breast cancer can be divided into four types; (1) luminal A, (2) luminal B, (3) basal-like, which is similar to triple-negative breast cancer (TNBC) and (4) human epidermal growth factor receptor (HER2). The cancer types are often associated with estrogen receptor (ER), progesterone receptor (PR), and HER2. The immunohistochemistry (IHC) of these receptor expressions like ER/PR positive or negative and HER2 positive or negative is used for the identification of the breast cancer subtypes [6]. The most aggressive type of breast cancer is TNBC and totals about 15–25% of all types. TNBC is difficult to treat because it does not have any effect on HER2-therapies and hormones as they lack expression of ER, PR, and HER2 receptors. Breast cancer is a systemic metastatic disease. Mortality due to breast cancer is high and is caused by metastasis [7].

World Health Organization (WHO) reported that there were about 2 million breast cancer incidents and 685,000 deaths globally by the year 2020. Prominent dangerous elements of breast cancer include aging, personal and family history of breast conditions and cancer, inborn genes (*BRCA1* and *BRCA2* have an increased risk; *PTEN*, *ATM*, *TP53*, *CHEK2*, *STK11*, and *PALB2* carry a low risk of breast cancer), radiation, and obesity. Estimated danger elements include nulliparous, single pregnancy, not breastfeeding, postmenopausal hormone therapy, oral intake of contraceptives, high fat intake, low fibers intake, liquor, and smoking (https://www.who.int/news-room/fact-sheets/detail/breast-cancer (accessed on 30 October 2021)). A case study in Mexico City reported that obesity and overweight and lactation have a high risk of causing breast cancer in women [8].

Scientists all over the world are involved in immense research to prevent and treat breast cancer. Due to the booming technological advancements, the development of synthetic medicines began and the usage of medicinal plants was forgotten for a long term. However, because of the severe side-effects caused by those synthetic medicines, the potentials of phytomedicine have received increased attention for the past few decades [9]. Plant extracts cause minimal side effects in comparison to chemotherapeutic drugs and therefore, nowadays, natural remedies are preferred [10]. Though natural remedies cannot cure breast cancer, maintaining a proper diet, regular exercise and good sleep will help to fight against cancer. Eating flavonoid-rich vegetables and fruits like onions, eggplant, garlic, potatoes, tomatoes, lettuce, peppers, apple, orange, and aromatic herbs can reduce the risk of breast cancer [11]. Plants have become a non-toxic and safe source of anticancer compounds. Scientific evidence supports that the extracts of various plants can induce apoptosis in cancer cells [12]. However, not all compounds in plants are non-toxic. Several compounds like ricin present in plants can cause severe toxicity [13]. Therefore, proper attention should be given to mining the potential compounds.

**Figure 1:**
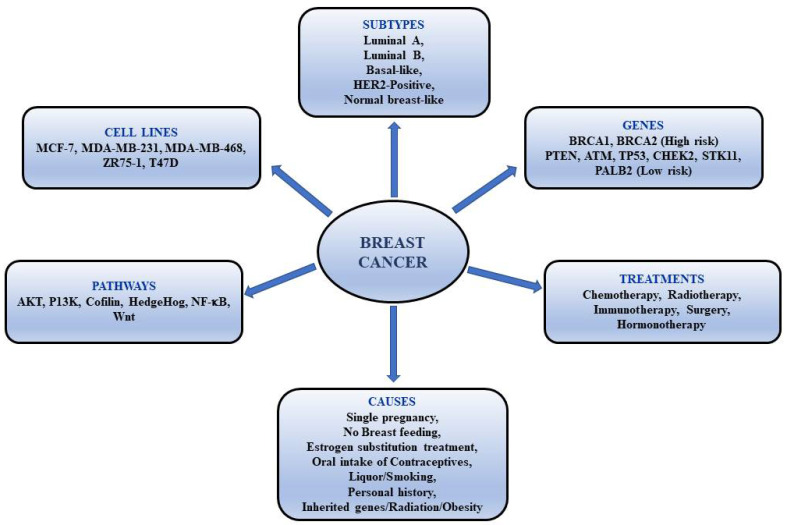
Breast cancer-types, genes involved, causes and cell lines.

European Code Against Cancer reported that about 9% of breast cancer is caused due to lack of physical activity [14]. The European Journal of Oncology Nursing reported that around three hours of exercise per week reduced the risk of patients suffering from breast cancer. Therefore, the risk of breast cancer can be reduced with routine exercise. Reducing body fat, especially after menopause, can also decrease the risk. Therapeutic approaches for breast cancer consist of surgery, hospitalization, radiation therapy, chemotherapy, and hormone therapy. On the other hand, bioactive compounds from plant source can be combined with chemotherapeutic drugs to minimize the side effects. Breast cancer types, genes involved, causes, and cell lines are illustrated in Figure 1.

Bioinformatics has emerged as a valuable technology in identifying potential molecules and toxic compounds. Bioinformatic techniques such as systems pharmacology and cheminformatics play an important role in the development of novel drug compounds from medicinal plants [15]. These techniques are used to study the interactions between plant compounds and disease-specific human targets, thus contributing significantly to drug discovery. Recent bioinformatics studies depicted the regulatory network between transcriptional factors and immune genes which was useful to understand the immune regulatory mechanisms behind breast cancer. Systems pharmacology and cheminformatics play a major role in drug targeting, finding allosteric binding sites understating, drug efficacy, and toxicity [16,17].

To develop alternative therapeutics for breast cancer, it is important to have deeper understanding on (1) the genes involved in various forms of breast cancer and their originating cell with an immunohistochemical classification of receptors (2) pathways of breast cancer—Akt, cofilin, Hedgehog, nuclear factor-κB (NF-κB), PI3K, PI3K-Akt, PI3K-Akt-mTOR, and Wnt (3) mechanisms and genes or molecules that are responsible for breast cancer brain metastasis, (4) chemoresistivity—mechanism and drug/gene, (5) anticancer activity of medicinal plants along with its efficiency, (6) *in silico* approaches—system pharmacology and cheminformatics, and (7) statistical analysis of breast cancer individually and in comparison with other cancers. 

Hence, this review focuses on the divergent aspects to develop therapeutics for breast cancer with special significance given to plant-based-medicinal compounds and bioinformatics. It also focuses on alternative methods and the most efficient ways to develop drugs for breast cancer with minimal side effects.

## 2. Sources and Methodology

The highly relevant articles were retrieved via meticulous search on the databases, Web of Science, Scopus, PubMed, PubChem and Google Scholar. The keywords and phrases were used for the search are “anticancer activity”, “medicinal plants”, “breast cancer”, “anticancer plants”, “breast cancer genes and chemoresistivity”, “in vitro and in vivo activities”. The relevant articles number finalized after collated article extraction and analysis through combination of aforementioned keywords and the inclusion criteria was 146. The inclusion criteria were based on the following criteria (i) reported traditional anti-cancerous activity of plants, (ii) reported anti-cancerous role of extract from medicinal plants. The inclusion criteria were implied for selecting particular anticancer plants and their bioactives are given in detail.

## 3. Subtypes of Breast Cancer

### 3.1. Luminal A

Luminal A is the common subtype of breast cancer that comprises around 50–60% of all cases. High ER level and low proliferation genes level are the attributes of luminal A. In addition, expression of cytokeratins [18,19], luminal-associated markers such as ER1, ER function-associated genes including hepatocyte nuclear factor 3 alpha (FOXA1) and B cell lymphoma 2 (BCL2) are the characterizations of Luminal-A [20]. Recent research has identified NCAM1 and NMUR1 as novel genes involved in luminal A cancer [21].

### 3.2. Luminal B

Luminal B is the most aggressive type and represents 15–20% of breast cancers [20]. High expression of proliferation-related genes such as, avian myeloblastosis viral oncogene homolog (ν-MYB), gamma-glutamyl hydrolase (GGH), lysosome-associated transmembrane protein 4-beta (LAPTMB4), nuclease sensitive element-binding protein 1 (NSEP1), and cyclin E1 (CCNE1) in luminal B is the main difference between both luminal subgroups [18].

### 3.3. Basal-like

Basal-like tumors represents 8–37% of all breast cancers [20,22]. Approximately 20–30% discordance is studied between triple-negative and basal-like. Triple-negative refers to the immunohistochemical classification of breast tumors that are lacking ER, PR, and HER2 protein expression, whereas the basal-like subtype is defined through gene expression microarray analysis [23]. Basal-like breast cancer possesses the most aggressive clinical features. Since there is no effective treatment including radiotherapy, TNBC is difficult to treat. It showed Keratin 5/6 and 17 and EGFR-positivity [24]. Jia et al., has reported that the gene STIL plays a significant role in the basal-like subtype [21].

### 3.4. HER2-Positive

The human epidermal growth factor receptor-2 belonging to tyrosine kinases family, located in chromosome 17q21 is encoded by the HER2 gene [19,25]. HER2-positive represents 15–25% of breast cancer subtypes and has aggressive biological and clinical behavior [25].

### 3.5. Normal Breast-like

Approximately 5–10% of all breast carcinomas are normal breast-like tumors. Since the expression of ER, PR, and HER2 are lacking in normal breast-like tumors, these can be classified as triple-negative and different from basal-like as they are CK5 and EGFR negativity [18,26]. The subtypes of breast cancer and its details are given in Table 1.

## 4. Pathways Involved in Breast Cancer

The Akt pathway, downregulated in various cancers, is a key regulator of cell multiplication and survival. Breast cancer tissue microarrays analysis showed that the Akt pathway is activated in the ductal carcinoma *in situ* (DCIS) stage, which also refers to stage 0 breast cancer [27]. Metformin decreases Akt activation by activating Adenosine 5′-monophosphate-activated protein kinase (AMPK), which leads to increased phosphorylation of IRS-1 at Ser^789^ [28].

The invasive and metastatic phenotype of tumor cells is determined by the overall activity of the cofilin pathway. Cofilin, a small ubiquitous protein, which is approximately 19 kDa, is found in invasive mammary tumor cells. The downregulation of cofilin is observed in breast cancer patients and also in individuals with mutations in the BRCA 1 tumor-suppressor gene [29].

The abnormal activation of the Hedgehog (Hh) signaling pathway is distinctly tied to cancer development and progression in a variety of solid malignancies. Hh ligand overexpression is associated with the basal-like subtype of breast cancer phenotype [30]. The Hh pathway plays an essential role in embryonic patterning, and it is also involved in stem cell renewal, tissue regeneration and repair. Three secreted ligands are involved in signaling cascade-sonic Hedgehog (SHH), Indian Hedgehog (IHH), and Desert Hedgehog (DHH). The Hh pathway activation enhances the proliferation invasion and migration of TNBC cells [31]. A steroidal alkaloid, cyclopamine blocks the Hh pathway, suppresses Gli1 expression, and thereby inhibits breast carcinoma cell growth [32]. It is also reported that the Hh pathway mediates the progression from DCIS to invasive ductal carcinoma (IDC) and the Gli1 nuclear translocation ratio could be used as a biomarker for evaluating the ability of invasiveness [33].

Nuclear factor-κB (NFκB) belongs to the family of ubiquitously expressed transcription factors is required for normal mammary gland development. NFκB is associated with the progress of ER-negative breast cancer. Clinical evidence reveals that the DNA-binding by p50 subunit of NFκB can be used as a prognostic marker for identifying a high-risk subset of ER-positive [34]. NFκB activity was inhibited by parthenolide (PTL), pyrrolidinedithiocarbamate (PDTC), and its analog diethyldithiocarbamate (DETC) in MCF7 sphere cells [35]. A natural isoflavonoid, Genistein, found in soybean products regulates the expression of genes that prompt apoptosis in breast cancer cells. The inactivation of NFκB by Genistein in MDA-MB-231 cancer cells is partially mediated through the Akt pathway [36]. 

The genetic and genomic studies have revealed new paths that are activated in various breast cancers, in which somatic mutations occur due to the gain or loss of key genes within the phosphoinositide 3-kinase pathway (PI3K) [37]. PI3K pathway was reported to have alterations in many cases of breast cancers and leads to therapeutic resistance. It is also reported that more than 70% of breast cancers have a modification in at least in one component of the PI3K pathway, which might be exploited to therapeutic advantage in “basal-like” cancers [38]. One of the most frequent oncogenic aberrations of TNBC is the dysregulation of signaling through the PI3K and Akt signaling pathways [39]. 

PIK3CA mutations and Akt activation by phosphorylation (pAkt) are commonly detected in various cancers, but its frequency is found to be high in breast cancer [40]. The preclinical and neoadjuvant trial data suggested that a PIK3CA alteration showed tolerance to HER2-targeted therapy. The validation of these alterations has treatment modalities towards further advancement of precision medicine for breast cancer [41]. The activation of the PI3K-Akt pathway through the loss of PTEN or PIK3CA mutation was frequently observed in trastuzumab-refractory human breast cancers [42].

PI3K-Akt-mTOR signaling pathway plays a significant role in cancer development because it is involved in regulation of cell growth and survival, apoptosis, motility, cell cycle, and various metabolic functions [43]. The mechanisms involved in activation of a PI3K-Akt are constitutively activated receptor tyrosine kinases (IGF/IGFR, ErbB, FGF/FGFR systems) leading to constitutive activation of PI3K; phosphatase and tensin homolog (PTEN) gene function, PI3K mutations: aberrant activation of Akt, eIF4E, 4E-BP1, and p70S6K, where these alterations trigger a cascade of biological events, i.e., from cell growth and proliferation to survival and migration, which contribute to tumor progression. Thus, this pathway is considered a target for the development of novel anticancer molecules [44]. In addition, alterations in the genes encoding several nodes of the PI3K-Akt-mTOR pathway is frequently found in ER-positive breast cancer, which includes activating mutations in the genes encoding IGF-1R and InsR, p110α PI3K, PDK1, HER2, and Akt1, and loss of expression of genes encoding PTEN [45].

The Wnt pathways have not only been generated in the context of cancer development but also in cancer pathogenesis and, thus, redefining cancer as a result of dysregulation of the developmental process. The types of Wnt pathway are canonical pathway and non-canonical pathway [46]. Wnt antagonists like APC, SFRP1/2, CDH1, and activator β-catenin (CTNNB1), along with the increased nuclear accumulation of β-catenin, played a crucial role in the prognosis of breast cancer and had significant clinical as well as prognostic importance [47]. Furthermore, TamR cells exhibited increased Wnt signaling when measured through TOP/FOP Wnt luciferase reporter assays. Genes associated with β-catenin dependent (AXIN2, MYC, CSNK1A1) and independent arms (ROR2, JUN) as well as Wnt secretion (PORCN) of the Wnt signaling pathway, were upregulated in TamR cells [48].

## 5. Breast Cancer Brain Metastasis

In recent years, the incidence of deaths due to breast cancer brain metastasis (BCBM) has increased. αB-crystallin gene (CRYAB), a molecular chaperone, was reported as the strongest independent predictor of BCBM, and it could be used as a biomarker to identify the patients with a high risk of breast cancer for early relapse in the brain [49]. The subpopulation of the cells that are present in BCBM circulating tumor cells can be used as a biomarker and also for making decisions about the treatment [50].

The breast cancer cell lines are attracted through chemokines CXCL16 and CXCL12 by fibroblasts that are associated with BCBM and therefore, blocking receptor-ligand interaction of CXCR6-CXCL16/CXCR4-CXCL12, which may be preventive therapy for BCBM [51]. Four miRNAs, miR-199A-5p, miR-132-3p, miR-155-5p and miR-150-5p, which were expressed between breast cancer that did not relapse (BCNR) vs. primaries that relapse (BCR) and primaries that relapse (BCR) vs. brain metastasis (BM), can be used to predict the survival of a patient with BCBM [52]. A major complication of breast cancer is blood-borne metastasis to the brain. The proliferation in the brain is caused due to increased Hypoxia Inducible Factor 1A (HIF1A)-associated signaling. Thus, the therapeutic implication may be used for the activation of hypoxic signaling [53]. Recent research hypothesized that nephronectin, an extracellular matrix protein promotes BCBM via integrin (α8β1) binding motifs [54]. The BCBM is promoted by the interaction of astrocytes in the brain and the invading triple-negative breast cancer cells via TGF-β2 (Transforming Growth Factor-beta-2) produced by astrocytes and is responsible for ANGPTL4 expression upregulation/angiopoietin-like 4 (ANGPTL4) [55]. The epithelial-to-mesenchymal transition, invasion, and BCM are inhibited by the GATA3-UTX-Dicer axis, where GATA3, a type of transcription factor, is positively correlated with a histone H3K27 demethylase, UTX [56]. The various mechanism of different genes or molecules and their role are mentioned in Table 2.

## 6. Chemoresistivity

Recent studies revealed that nitrogen had the efficiency to block tumor metastasis of TNBC by promoting mesenchymal to epithelial transformation in MDA-MB-231-Luciferase cells [57]. TXX-1-10, a derivative of rimonabant, reduced expression of HPIP and has an inhibitory effect on breast cancer growth and metastasis [58]. Trifluoperazine hydrochloride, an antipsychotic drug, is administered to suppress the growth of TNBC and brain metastasis by inducing apoptosis and G0/G1 arrest via reducing the cyclin D1/CDK4 and cyclin E/CDK2 expressions in MDA-MB-486, MDA-MB-231, and 4T1 cancer cell lines [59]. A transcription suppressor and a significant proto-oncogene, the factor that binds to the inducer of short transcript-1 (FBI-1) increased the expression of PXR by inhibiting miR-30c expression [60]. Thus, FBI-1 mediates drug resistance of TNBC cells through miR-30c/PXR axis. The zinc finger E-box binding homeobox 1 (ZEB1) expression showed a positive correlation with the expression of Bcl-xL and cyclin D1 [61]. Additionally, the ataxia-telangiectasia mutated (ATM) was transcriptionally activated by ZEB1 and formed ZEB1/p300/PCAF complex, which mediates DNA damage repair and clearance of DNA breaks. It showed that ZEB1 was a determinant of chemoresistance in breast cancer [61]. Cell cycle-related E3 ubiquitin ligase checkpoint with fork-head and ring finger domains (CHFR) plays a major role in the negative regulation of ZEB1. It has been inferred that CHFR-ZEB1 signaling acted as chemo resistive in malignant breast cancers [62]. The synergistic effect of melatonin and doxorubicin induces apoptosis by decreasing AMP-activated protein kinase α1 (AMPK α1) expression at the transcriptional level through an autophagy-dependent mechanism [63]. An actin-bundling protein fascin increased chemoresistance through P13K/Akt signaling and suppressed pro-apoptotic markers such as caspase 9, caspase 3, and PARP, which resulted in regulation of breast cancer metastasis and survival [64]. Luteinizing Hormone–Releasing Hormone (LHRH) is conjugated with prodigiosin (PGS) and paclitaxel (PTX) inhibited the growth of TNBC in in vitro and in vivo experiments [65]. The mechanism of various types of drugs/genes are shown in Table 3.

## 7. Anticancer Activity of Medicinal Plants

Phytochemicals can be used as chemotherapeutics that are isolated from various plant extracts as these phytochemicals have shown diverse effects on anti-tumor, anti-inflammation, anti-oxidant, and anti-bacterial [66]. Phytochemicals such as vinca alkaloids, taxanes, Camptothecin derivatives, Cephalotaxus, Colchicine, Ellipticine, Berberine, Combretastatins, and triterpenoid acids showed the anticancer activity against various cancer types [67]. Plants also have positive results in curing diabetes, sterility, thyroid, fertility, and physiological disorders [68].

### 7.1. Echinacea

*Echinacea* belongs to the family *Asteraceae. E. purpurea, E. angustifolia, E. pallida* are the most commonly used species. *E. purpurea* is most commonly used in cancer treatment. It is commonly known as coneflower. It increases the number of Natural Killer Cells (NKCs), and the flavonoids act as an immune stimulant [69]. The crude extracts of root and leaf of *E. purpurea* possess IC_50_ values of 350 μg/mL and 280 μg/mL against BT-549 cell lines [70]. *E. angustifolia* DC extract was obtained with ethyl acetate (*Ea*-AcOEt). When this extract was quantified with HPLC, echinacoside and caffeic acid content was assessed and its cytotoxicity against MDA-MB-231 and MCF-7 cells were found to be 28.18 ± 1.14 μg/mL and 19.97 ± 2.31 μg/mL, respectively. In addition, *Ea*-AcOEt showed a synergistic effect with paclitaxel [71].

### 7.2. Allium sativum

*Allium sativum* is commonly known as garlic. Allicin, the originator of the sulfur-containing compound, is responsible for therapeutic properties. Another sulfur-holding substance called Ajoene delays the development of cancer. Selenium acts as an antioxidant. Ripened extract of garlic helps in shielding the propagation of cancers [72]. Ajoene restrained the growth of human breast cancer cells [73]. *A. sativum* exhibits anticancer activity against MCF-7 breast cancer cells when encapsulated with silver nanoparticles and had showed an IC_50_ value of 89.86 μg/mL [74].

### 7.3. Curcuma longa

*Curcuma longa*, which is commonly known as turmeric, has an ingredient called curcumin, which has anticancer activity due to the presence of phenolic substances [75]. Curcumin-loaded nanoparticles have low toxicity and anti-inflammatory effect. It induced the inhibition of NFκB, AP-1, and STAT3 transcription factors and it also arrested cell cycle in the G2/M phase [76]. Further, pulsed electric field (PEF) treated C. longa showed effective inhibition against MCF-7 breast cancer cell line, and this could be an alternative method to treat cancer with minimal side effects [77]. The ethanolic extract of *C. longa* had IC_50_ values of 49 ± 2.08 μg/mL in 0.25% DMSO and 40 ± 1.03 μg/mL in 0.5% DMSO against MDA-MB-231 cell line [78]. 

### 7.4. Arctium lappa

*Arctium lappa* is commonly known as Burdock. Its seeds contain Arctigenin, which is one of the potential anti-cancerous compounds and it can eliminate tumor cells and their formation [79]. Lappaol F, an anticancer agent which has been isolated from *Arctium lappa* L. has arrested the G2 cell cycle by inducing the G1 phase. p21 plays a vital role in G2 arrest by lappaol F-mediated regulation of CDK1 and cyclin B1 [80]. The synergetic effect of *A. lappa* root extract and doxorubicin showed apoptotic effect and antiproliferative effect against MCF-7 and MDA-MB-231 cell lines [81]. It also exhibits an IC_50_ value of 41.5 μmol/L for the MDA-MB-231 cell line [82]. 

### 7.5. Synadenium cupulare

*Synadenium cupulare* leaf water extract was used for the synthesis of CdO/CdO_3_ nanocomposite using cadmium nitrate tetrahydrate. Tannins, saponins, flavonoids and glycosides were present in the leaf extract which showed anti-cancer properties. The IC_50_ values of various extracts are shown in Table 4 [83].

### 7.6. Cimicifuga foetida

*Cimicifuga foetida* is commonly known as black cohosh. Actein, a triterpene glycoside inhibits Ras/MEK/ERK signaling pathway and Akt phosphorylation in MCF-7 cells and suppresses TNFα-induced IKKα/β and IKKα phosphorylation, as well as NFκB downstream targeting gene expression in TNBC [84]. A cycloartane triterpenoid, KHF16 which was isolated from rhizomes of *C. foetida* induced G2/M phase arrest and apoptosis in MDA-MB-468 and possess IC_50_ of 5.6 μM, 6.8 μM, and 9.2 μM against MCF-7, MDA-MB-231, and MDA-MB-468 cell lines, respectively [85].

### 7.7. Cymbopogon citratus

*Cymbopogon citratus* is a citrus-based plant, which contains citral. Nanostructured Lipid Carrier–citral showed better results than citral itself when treated in MDA-MB-231 breast cancer cells [86]. Essential oil of *C. citratus* showed anticancer effect on 7, 12-Dimethylbenz—[α]-anthracene (DMBA)-induced breast cancer in female rats with IC_50_ values of 12.03 μg/mL [87]. Furthermore, polysaccharide fractions of *C. citratus* showed cytotoxic and apoptotic effects by upregulating caspase3, downregulating bcl-2 genes followed by the release of cytochrome C [88].

### 7.8. Zingiber officinale

*Zingiber officinale* is commonly kwon as Ginger and is widely used for the treatment of cancer [89]. The extracts of *Z. officinale* possess significant antiproliferative activity against TNBC [90]. In addition, *Z. officinale* suppresses MCF-7 and MDA-MB-231 cell lines proliferation and colony formation by upregulating Bax and downregulating Bcl2 proteins. It also downregulates the expression of NF-κB, Bcl-X, Mcl-1 and Survivin, cyclin D1, and CDK-4 [91]. Fulbaria and Syedpuri, the two Bangladeshi ginger varieties and showed anticancer activity in MCF-7 and MDA-MB-231 cell lines with IC_50_ values of 34.8 and 25.7 μg/mL for Fulbaria. MDA-MB-231 exhibition were 32.53 and 30.20 μg/mL for rhizomes extract of Fulbaria and Syedpuri [92].

### 7.9. Rhus coriaria

*Rhus coriaria*, known as Sumac, contains phenolic acids, flavonoids, and tannins [93]. It suppresses the metastasis of breast cancer by inhibiting STAT3, NKκB, and NO pathways. *R. coriaria* ethanolic extract (RCE) inhibits cell proliferation by cell cycle arrest at the G1 phase and senescence [94]. *R. coriaria* was evinced to be a promising therapeutic drug against breast cancer as it induced senescence and autophagy cell death by the activation of p38 and ERK1/2 [95]. RCE also upregulates p21, downregulates cyclin D1, c-myc, PCNA, p27, phosphor-RB, and senescence-associated β-galactosidase expressions in T47D, MCF-7, and MDA-MB-231 cell lines. MDA-MB-231 cell lines showed an IC_50_ value of 215 μg/mL whereas MCF-7 cell lines showed 155 μg/mL [96]. 

### 7.10. Ricinus communis *L.*

*Ricinus communis* L. fruit extract showed antiproliferative activity on MCF-7, MDA-MB-231. It contains various medicinally important compounds such as Ricinine, p-coumaric acid, Epigallocatechin, and Ricinoleic acid [97]. The phytochemicals present in *R. communis* target peroxisome proliferator-activated receptor (PPAR), NF-κB, cytochrome p450, p38 MAPK, p53, and Bcl-xL [98]. The seed of *R. communis* has been encapsulated with zinc oxide nanoparticles and it was reported that IC_50_ against MDA-MB-231 cells was 7.103 μg/mL [99].

### 7.11. Drosera bormannii

*Drosera bormannii*, commonly known as the Sundew plant, has anti-inflammatory and anti-cancer activity. It arrests the cell cycle at the G2/M phase and induces apoptosis in MCF-7 cells. It also upregulates the p53 and Bax/Bcl ratio. Compounds present in *D. bormannii* includes hexadecenoic acid, tetradecanoic acid, hexadecen-1-ol, trans-9 and 1-tetradecanol, and other fatty acids. The IC_50_ value was found to be 120.94 ± 1.91 μg/mL when MCF-7 cells have been treated with methanolic extract of *Drosera bormannii* [100]. 

### 7.12. Acacia hydasica

*Acacia hydasica* R. parker contains four active phenolic compounds namely catechin, 7-O-galloyl catechin, catechin-3-O-gallate (CG), methyl gallate (MG). MDA-MB-231 breast cancer cell growth was inhibited by MG and CG. It also induced the reduction of CK2α, Bcl-XL, surviving, and xIAP protein expression through the suppression of the JAK/STAT pathway, NKκB, and p13K pathway [101].

### 7.13. Saussurea lappa

*Saussurea lappa* root contains two natural sesquiterpene lactones namely costunolide (cos) and dehydrocostuslactone (Dehy). The apoptotic activity of breast cancer is inhibited through AKT/14-3-3 and c-Myc/p53 pathway [102]. The dried roots of *S. lappa* contain sesquiterpene lactone that acts as an anti-tumor agent against the MCF-7 cell line with an IC_50_ value of 35.05 ± 9.37 μg/mL [103]. Saussurea lappa Clarke (SLC) and its derivative costunolide have been reported to suppress MDA-MB-231 cell growth and metastasis by inhibiting TNF α-induced NF-κB activation [104].

### 7.14. Centella asiatica

*Centella asiatica* possess anticancer activity against MCF-7 cell lines [105]. It contains Asiatic acid which inhibits WAWE3 expression and activation through the P13K/Akt pathway-invasion and proliferation of MDA-MB-231 cells [106]. Aqueous extract of *C. asiatica* have exhibited the IC_50_ of 648.00 μg/mL against MDA-MB-231 cell lines [107].

### 7.15. Eclipta alba

Wedelolactone, a compound present in *Eclipta alba* inhibits MCF-7, T47D, and MDA-MB-231 cells by stimulating ER signaling [108]. Chloroform fractionization of *E. alba* leaves disrupts the mitochondrial membrane potential, upregulates Hsp60, and downregulates the expression of anti-apoptotic protein XIAP as it activates an apoptotic pathway in MCF-7, MDA-MB-231. The IC_50_ values were found to be 18.03 ± 2.0 μg/mL and 42.5 ± 3.5 μg/mL against both cell lines [109]. The list of medicinal plants and their extract/compound that acts on various cell lines with their IC_50_ values are summarized in Table 4.

**Table 4:** Medicinal plants and its extract/compound that acts on various cell lines with its IC_50_ values.

Plant	Structure	Extract/Compound	Cell Lines	IC_50_	References
*Echinacea purpurea*	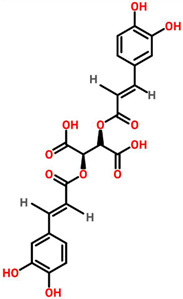 (Chicoric acid)	Crude extract of root	BT-549	350 μg/mL	[70,110]
		Leaf extract	BT-549	280 μg/mL	
*Echinacea angestifolia*	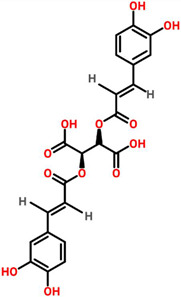 (Chicoric acid)	*EA*-AcOEt	MDA-MB-231	28.18 ± 1.14 μg/mL	[71,110]
			MCF-7	19.97 ± 2.31 μg/mL	
*Allium sativum*	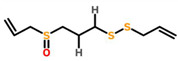 (Ajoene)	*A. sativum* fruit extract encapsulated with silver nanoparticles	MCF-7	89.86 μg/mL	[74]
*Curcuma longa*	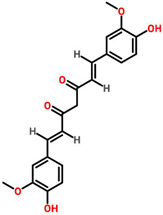 (Curcumin)	Ethanolic extract	MDA-MB-231	49 ± 2.08 μg/mL (0.25% DMSO)	[78]
				40 ± 1.03 μg/mL (0.5% DMSO)	
*Arctium lappa*	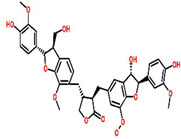 (Lappaol F)	Lappaol F	MDA-MB-231	41.5 μmol/L	[82]
*Synadenium Cupulare*	--	Hexane extract	MCF-7	1.427 ± 0.612 μg/mL	[83]
			MDA-MB-231	36.58 ± 3.54 μg/mL	
		DCM extract	MCF-7	0.202 ± 0.612 μg/mL	
			MDA-MB-231	9.716 ± 3.06 μg/mL	
		Methanol extract	MCF-7	45.71 μg/mL	
			MDA-MB-231	Above 100 μg/mL	
		Ethyl acetate extract	MCF-7	58.71 μg/mL	
		Annealed CdO/CdCO3 nanocomposite	MCF-7	0.652 ± 2.532 μg/mL	
			MDA-MB-231	Above 100 μg/mL	
		Unannealed CdO/CdCO3 nanocomposite	MCF-7	3.770 ± 0.530 μg/mL	
			MDA-MB-231	3.088 ± 0.637 μg/mL	
*Cimicifuga foetida*	--	Cycloartane triterpenoid, KHF16 isolated from rhizomes	MCF-7	5.6 μM	[85]
			MDA-MB-231	6.8 μM	
			MDA-MB-468	9.8 μM	
*Cymbopogon citratus*	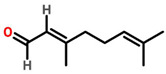 (Citral)	Essential oil	DMBA-induced breast cancer in female rats	12.03 μg/mL	[87,111]
*Zingiber officinale*	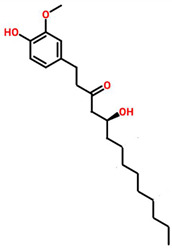 (10 Gingeral)	Fulbaria	MCF-7	34.8 μg/mL	[91,112]
			MDA-MB-231	25.7 μg/mL	
		Fulbaria (Rhizome extract)	MDA-MB-231	32.53 μg/mL	
		Syedpuri (Rhizome extract)	MDA-MB-231	30.20 μg/mL	
*Rhus coriaria*	--	Ethanolic extract	MCF-7	155 μg/mL	[96]
			MDA-MB-231	215 μg/mL	
*Ricinus communis L.*	--	Seed extract encapsulated with zinc oxide nanoparticles	MDA-MB-231	7.103 μg/mL	[99]
*Drosera burmannii*	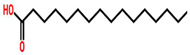 (Palmitic acid) 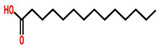 (Myristic acid) 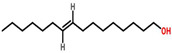 (Hexadecen-1-ol,trans-9-) 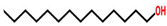 (1-tetradecanol)	Methanolic extract	MCF-7	120.94 ± 1.91 μg/mL	[100]
*Acacia hydasica*	--	Phenolic	MDA-MB-231	--	
*Saussurea lappa*	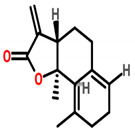 (Costunolide) 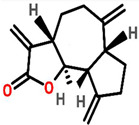 (Dehydrocostunolide)	Sesquiterpene lactone isolated from dried roots	MCF-7	35.05 ± 9.37 μg/mL	[103]
*Centella asiatica*	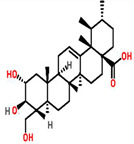 (Asiatic acid)	Aqueous extract	MDA-MB-231	648.00 μg/mL	[107]
*Eclipta alba*	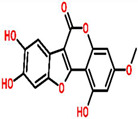 (Wedelolactone)	Leaf extract	MDA-MB-231	42.5 ± 3.5 μg/mL	[109]
			MCF-7	18.03 ± 2.0 μg/mL	

## 8. Bioinformatics Approaches

### 8.1. Systems Pharmacology

One of the most prominent emerging tools to study the interactions between drugs and biological systems is systems pharmacology [113]. Systems pharmacology, also known as network pharmacology, can be used to find the predictive targets, protein-to-protein interaction networks, and signaling pathways in drug treating diseases [114]. In recent years, various natural products were identified using systems pharmacology along with their mechanisms [115]. Subsequently, network biology also plays an important role in the identification of significant genes associated with various diseases.

Recent studies revealed that the systems pharmacology approach identified wogonoside as an effective angiogenesis inhibition in TNBC [116]. A systems pharmacology approach was employed between the major compounds of Iranian *Chrysanthemum* cultivars and known breast cancer drugs with breast cancer-related targets to analyze the mechanism. Among the major compounds of these cultivars, rutin has shown anticancer activity against MCF-7 cell line [117]. The systems pharmacology approach gives an idea of the target compound network as well as the signaling pathways associated with treating complex diseases like breast cancer apart from the confirmation study of the chosen target [118].

Quantitative systems pharmacology (QSP) bridges systems biology with pharmacokinetics (PK) and pharmacodynamics (PD), which gives a maximum understanding of the drug’s efficacy and toxicity in complex disease systems like breast cancer [119]. Recent research demonstrates the possibility of combining single-cell data to initialize cell states in special quantitative systems pharmacology (SPQSP) for the prediction of TNBC immunotherapy response [120].

Immune oncology also known as cancer immunotherapy is a form of cancer treatment that directs the patient’s own immune system to fight against cancer. Recently developed QSP modes will be increasing by use in IO drug development, which will become IO practice to run virtual trials along with critical trials on the other hand. This can quickly bring better therapeutics to cancer patients [121].

### 8.2. Cheminformatics

The substantial growth in epigenetics-related data in recent years has led to the development of cheminformatics methods. Hidden allosteric binding sites and protein–protein interaction hotspots for epigenetic targets could be found using computational approaches. In addition, molecular modeling and cheminformatics have made significant contributions to drug discovery [122]. Quantitative structure–activity relationship (QSAR) is also one of the most important methods used for drug discovery. This employs a multi-target approach that can be used to predict anti-cancer agents against various cell lines simultaneously.

Cheminformatics-based selection of small molecules binary weapons that improve transporter-mediated targeting helps in enhancing drug efficacy and therapeutic index [123]. Cheminformatic approaches along with web ontology language can be used for exploring the pharmacogenomics knowledge base for repositioning breast cancer drugs, which gives better performance of new indications and possible conflicting effects prediction for breast cancer drugs. Drug repurposing is one of the most efficient approaches to speed up the drug discovery process by finding new therapeutic uses from existing drugs [124]. The important criteria for repurposing it is to understand and identify the relationship between diseases and drugs, which can be achieved with the help of cheminformatic approaches. Computational approaches that use cheminformatics and molecular modeling methods have been reported to speed up natural product-based drug discovery. Organization, analysis, and dissemination of chemical information of the natural products in compound databases are some of the applications of cheminformatics. Other applications are computer-aided natural product selection, identification of molecular targets for natural products, de novo design, and quantification of natural product likeliness [125].

## 9. Recent Trends in Indigenous Medicinal Plant Informatics and Avenues to Combat Cancer

With the advent of bioinformatics, information technology and omics, there is an ever-increasing trend to build resources and knowledgebases that reports herbal formulations, bio-active compounds of the medicinal plants and related information. There are several efforts such as Indian Medicinal Plants, Phytochemistry And Therapeutics (IMPPAT) [126], SymMap [127], Indian medicinal plants database (IMPLAD) [128], Collective Molecular Activities of Useful Plants (CMAUP) [129], etc. Furthermore, researchers have developed the novel strategies for *in silico* based pharmacokinetic properties of drugs/bioactive molecules [130,131,132,133,134]. These approaches are also applicable foe phytochemicals and plant bioactive molecules for their virtual screening, plausible and possible mode of mechanism and drug discovery [135]. Many plant-based anti-cancerous bioactive molecules have been evaluated via computational biology and systems pharmacology tools [136,137,138]. This review encourages further research on anticancer notably breast cancer active molecules for their bioinformatic screening and pharmacokinetic activities. Considering these facts, the plant derived bio-actives based drug formulations usually consist of many phytocompounds or even more than one particular plant. The main task on this path would be able to impute the role of phytochemicals other than active molecules and which are present in the traditional medicine.

## 10. Bioactive Compounds and Their Future Perspectives:

Medicinal plants contain several bioactive compounds that can be curative for various diseases, including cancer [139]. There are various traditional treatment methodologies in the world among which Ayurveda is the most commonly followed system in India. Some of the traditionally used plants against cancer in India includes Nichinda (*Vitex trifolia*), Indian Ipecac (*Tylopora indica*), Arjuna Bark (*Terminalia arjuna*), and other plants as previously mentioned in this review. These ethnobotanical resources can be used as natural alternatives because of their biomedical properties [140]. The secondary metabolites present in the plants are used for the production of drugs [141]. Apart from increasing the efficacy of chemo drugs, plant-based bioactive compounds can sometimes cause side effects when administered as chemotherapeutic agents [142]. These bioactive compounds also alter the biological pathways and modulate the immune system, which results in the suppression of breast cancer [143]. The expression of miRNAs can be changed using plant-derived bioactive compounds and thus it can be used as a promising approach for breast cancer treatment [144]. Bioactive compounds like tanshinones, berberine, matrine, and astragaloside IV showed inhibitory effect on breast cancer cells by rescuing miRNA expression [145]. These plants not only prevent or inhibit cancer, but also help in overall improvement of health.

## 11. Conclusions and Future Perspectives

As breast cancer is one of the leading causes of death observed in women, it is necessary to diagnose and treat it in the early stages to prevent metastasis. Since radiotherapy, chemotherapy, and surgery cause side effects, medicinal plants are used as a better alternative for its treatment. This holistic review details the different medicinal plants and their anti-cancerous potential. Not all the medicinal plants completely cure the disease; such plants are synthesizing the active anti-cancerous compounds. These compounds act synergistically with chemotherapeutic drugs to enhance the efficiency of the drug with minimal side effects. This review article also highlights the mechanism of antitumor action of some important native medicinal plants. It is generally done via diverse signaling pathways. These research analyses are notably performed in human cell lines. In addition, bioinformatics tools also play a key role in drug targeting, protein–protein interaction studies, identification of compound target networks, and pathways associated with treating complex diseases like cancer. QSP modes could be used in IO drug development that would help to run virtual trials on one hand and clinical trials on the other hand. This helps in bringing better therapies for cancer patients. *In silico* approaches are cost-effective and the results will be more precise so that this could be used as a tool to analyze the cancer-related target mechanism and to design drugs with more efficiency. Though, the analyses of these plants should not limit the study of plethora of anticancer medicinal plants, many of which remain unexplored. Further studies are required to highlight the possible or plausible mode of action along with *in silico* omics approaches of several explored and unexplored medicinal plants.

## Figures and Tables

**Table 1:** Details of subtypes of breast cancer.

Subtypes	Status of ER, PR & HER2	Originating Cell	Features of IHC	Percentage of Occurrence	References
Luminal A	ER+ or PR+ or Both, HER2-	Luminal Epithelial cell	Keratin 8/18 Positive	50–60%	[20]
Luminal B	ER+ or PR+ or Both, HER2+	Luminal Epithelial cell	Keratin 8/18 Positive	15–20%	[20,24]
Basal like	ER- or PR- or HER2±	Basal or Biopotent progenitor/myoepithelial cell	Keratin 5617 positive, EGFR positive	8–37%	[20,24]
HER2+	ER-, PR-, HER2+	Late luminal progenitor	----	15–25%	[20,24]
Normal breast like	Tumors do not fit into any of these categories	Luminal Epithelial cell	----	5–10%	[20,24]

**Table 2:** Various mechanism of genes or molecules and its role.

Genes or Molecules	Mechanism	Role	References
αB-crystallin Gene	Intracrine VEGF signaling and implicate UPR/CRYAB as dichotomous parts of regulation pathway	Biomarker	[49]
Subpopulation of BCBM CTCs	Inhibition of EIF2, IGF-, ILK, VEGF and Integrin signaling	Biomarker	[50]
Cancer-associated Fibroblasts	Chemokines CXCL16 and CXCX12 by fibroblasts and blocks the interaction of CXCR6-CXCL6/CXCR4-CXCL12	Preventive therapy for BCBM	[51]
miR-132-3p, miR-199A-5p, miR-150-5p and miR-155-5p	cMET-targeting	Predict the survival rate of patients and biomarker	[52]
Circulating Tumour Cells (CTCs)	Hypoxia Inducible Factor 1A-assocated signaling	Therapeutic implication	[53]
Nephronectin	Promotes BCBM via αβ1-binding motif	Reduced endothelial adhesion and transmigration	[54]
Interaction of astrocytes and invading TNBC cells	TGF-β2/ANGPTL4 axis	Promoting BCBM and ANGTL4 for treatment of BCBM	[55]
GATA3-UTX-Dicer axis	GATA3 expression is positively correlated with UTX, histone H3K27 demethylase	Epithelial-to-mesenchymal transition, invasion and BCM inhibition	[56]

**Table 3:** Drug/gene and its mechanism.

Drug/Gene	Mechanism	References
Nitrofen	Mesenchymal-to-epithelial transformation	[57]
TXX-1-10	Reduced the expression of HPIP	[58]
Trifluoperazine hydrochloride	Induce apoptosis and G0/G1 cell arrest by decreasing cyclin D1/CDK4 and cyclin E/CDK2 expression	[59]
FBI-1	Drug resistance of TNBC cells through miR-30c/PXR axis	[60]
ZEB1	ZEB1/p300/PCAF complex which mediates clearance of DNA breaks and DNA damage repair	[61]
	CHFR plays a major role in negative regulation	[62]
Synergistic effect of melatonin and doxorubicin	Apoptosis induction by (AMPK α1) at transcription level	[63]
Fascin	Chemoresistance through P13K/Akt signaling and suppressed proapoptotic markers	[64]
LHRH conjugation of PGS and PTX	Inhibition of TNBC growth	[65]
